# Novel Epigenetic Modulation Chitosan-Based Scaffold as a Promising Bone Regenerative Material

**DOI:** 10.3390/cells11203217

**Published:** 2022-10-13

**Authors:** Teerawat Sukpaita, Suwabun Chirachanchai, Theerapat Chanamuangkon, Katanchalee Nampuksa, Naruporn Monmaturapoj, Piyamas Sumrejkanchanakij, Atiphan Pimkhaokham, Ruchanee Salingcarnboriboon Ampornaramveth

**Affiliations:** 1 Center of Excellence on Oral Microbiology and Immunology, Faculty of Dentistry, Chulalongkorn University, Bangkok 10330, Thailand; 2 Center of Excellence on Petrochemical and Materials Technology, Chulalongkorn University, Bangkok 10330, Thailand; 3 Bioresources Advanced Materials, The Petroleum and Petrochemical College, Chulalongkorn University, Bangkok 10330, Thailand; 4 Biomaterial Testing Center, Faculty of Dentistry, Chulalongkorn University, Bangkok 10330, Thailand; 5 Assistive Technology and Medical Devices Research Center, National Science and Technology Development Agency, Pathum Thani 12120, Thailand; 6 Department of Anatomy, Faculty of Dentistry, Chulalongkorn University, Bangkok 10330, Thailand; 7 Department of Oral and Maxillofacial Surgery, Faculty of Dentistry, Chulalongkorn University, Bangkok 10330, Thailand; 8 Department of Microbiology, Faculty of Dentistry, Chulalongkorn University, Bangkok 10330, Thailand

**Keywords:** chitosan, biphasic calcium phosphate, trichostatin A, epigenetic, bone regeneration

## Abstract

Bone tissue engineering is a complicated field requiring concerted participation of cells, scaffolds, and osteoactive molecules to replace damaged bone. This study synthesized a chitosan-based (CS) scaffold incorporated with trichostatin A (TSA), an epigenetic modifier molecule, to achieve promising bone regeneration potential. The scaffolds with various biphasic calcium phosphate (BCP) proportions: 0%, 10%, 20%, and 40% were fabricated. The addition of BCP improved the scaffolds’ mechanical properties and delayed the degradation rate, whereas 20% BCP scaffold matched the appropriate scaffold requirements. The proper concentration of TSA was also validated. Our developed scaffold released TSA and sustained them for up to three days. The scaffold with 800 nM of TSA showed excellent biocompatibility and induced robust osteoblast-related gene expression in the primary human periodontal ligament cells (hPDLCs). To evaluate in vivo bone regeneration potential, the scaffolds were implanted in the mice calvarial defect model. The excellent bone regeneration ability was further demonstrated in the micro-CT and histology sections compared to both negative control and commercial bone graft product. New bone formed in the CS/BCP/TSA group revealed a trabeculae-liked characteristic of the mature bone as early as six weeks. The CS/BCP/TSA scaffold is an up-and-coming candidate for the bone tissue engineering scaffold.

## 1. Introduction

Bone is a dynamic organ composed of cortical and cancellous parts with remarkable regenerative properties [1,2,3]. Recent advancements in bone tissue engineering (BTE) and 3-dimension (3D) porous scaffolds have been used in bone defect repair for many years [4]. The development of BTE materials requires the continuous improvement of porous bone scaffolds with appropriate mechanical properties and suitable porosity to promote cell attachment and proliferation [5]. Moreover, incorporating growth factor molecules could even facilitate quality bone formation [6,7,8].

To develop bone substitute material with high biocompatibility and excellent osteogenic inducing capability, chitosan, a copolymer derived from chitin’s deacetylation, has been widely used as a bone scaffold because of its excellent biocompatibility and low toxicity [9,10,11,12]. However, the mechanical properties of these polymer scaffolds do not provide sufficient structural support [13,14], especially in the area needed for implant placement. To enhance the mechanical properties of chitosan for bone tissue engineering applications, several studies have tried to incorporate bio-ceramic into the chitosan scaffolds [15,16]. One of the most interesting bio-ceramics is BCP, which comprises a mixture of hydroxyapatite and β-tricalcium phosphate [17,18]. As a result, BCP exhibits good bioactivity and optimum degradation rates under the studied physiological conditions [19]. Moreover, the bone formation potential of chitosan composite scaffold can still be improved by incorporating additional osteoinductive biomolecules.

In the context of molecular biology, deoxyribonucleic acid (DNA) of mammalian cells regulates their function partly by changing their three-dimensional structure, coiling, and uncoiling, by wrapping twice around a histone octamer to render a nucleosome [20]. Two distinctive groups of enzymes control acetylation and deacetylation of histones and a few non-histone proteins. The acetylation and deacetylation process of histone proteins largely affect their interaction with DNA and alter the transcription of genes residing in the corresponding region of DNA. Histone acetylation, which opens up the chromatin structure, is catalyzed by histone acetyltransferase (HAT). At the same time, deacetylation results in a condensation of chromatin are influenced by histone deacetylase (HDAC) [21,22]. TSA is a broad-spectrum HDAC inhibitor, inhibiting histone deacetylase of several classes [23,24]. TSA has been reported to induce accumulation of the acetylated histone tail, which renders a loosening euchromatin structure that is more accessible for the transcriptional complexes and then turns on the transcription of genes in mammalian cells. Several previous studies have demonstrated that TSA specifically induces in vitro osteoblast differentiation and increases in vivo bone regeneration [25,26,27,28]. Huynh et al. reported previously that TSA was able to inhibit HDAC activity-induced hyperacetylation of Histone H3, demonstrating Histone H3 as a candidate target molecule for HDAC inhibition [29]. Therefore, TSA has been proven as an excellent candidate therapeutic agent to incorporate into a 3D porous scaffold for bone tissue engineering applications [26,30].

This study aimed to develop a chitosan biphasic calcium phosphate composite scaffold incorporated with trichostatin A (CS/BCP/TSA scaffold) and further examine their mechanical and biological properties both in vitro and in vivo experiments.

## 2. Results

### 2.1. Selection of Optimal Physicochemical Scaffold

**Figure 1:**
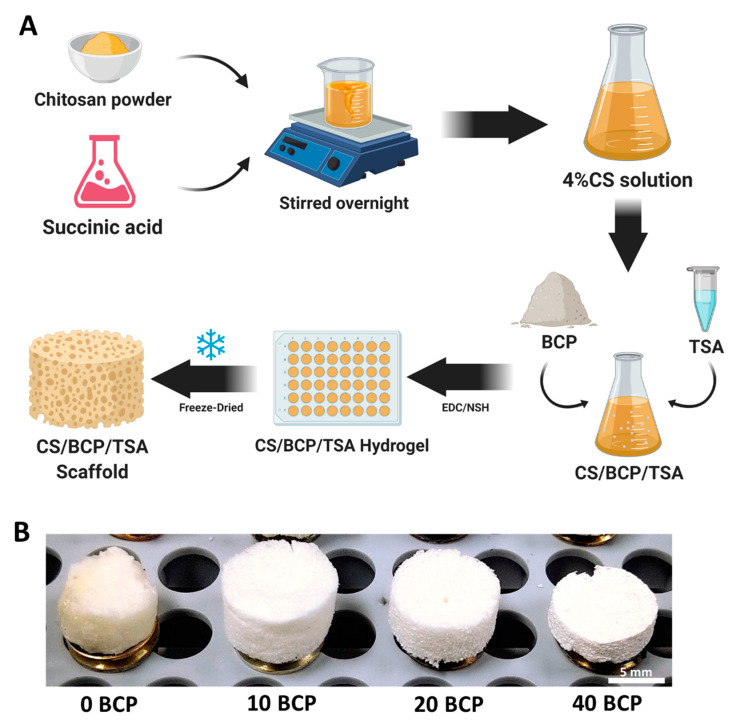
CS/BCP/TSA scaffold fabrication. (**A**) A schematic illustration of scaffold fabrication with freeze-drying technique. (**B**) Scaffold architectures: 0% BCP, 10% BCP, 20% BCP, and 40% BCP.

**Figure 2:**
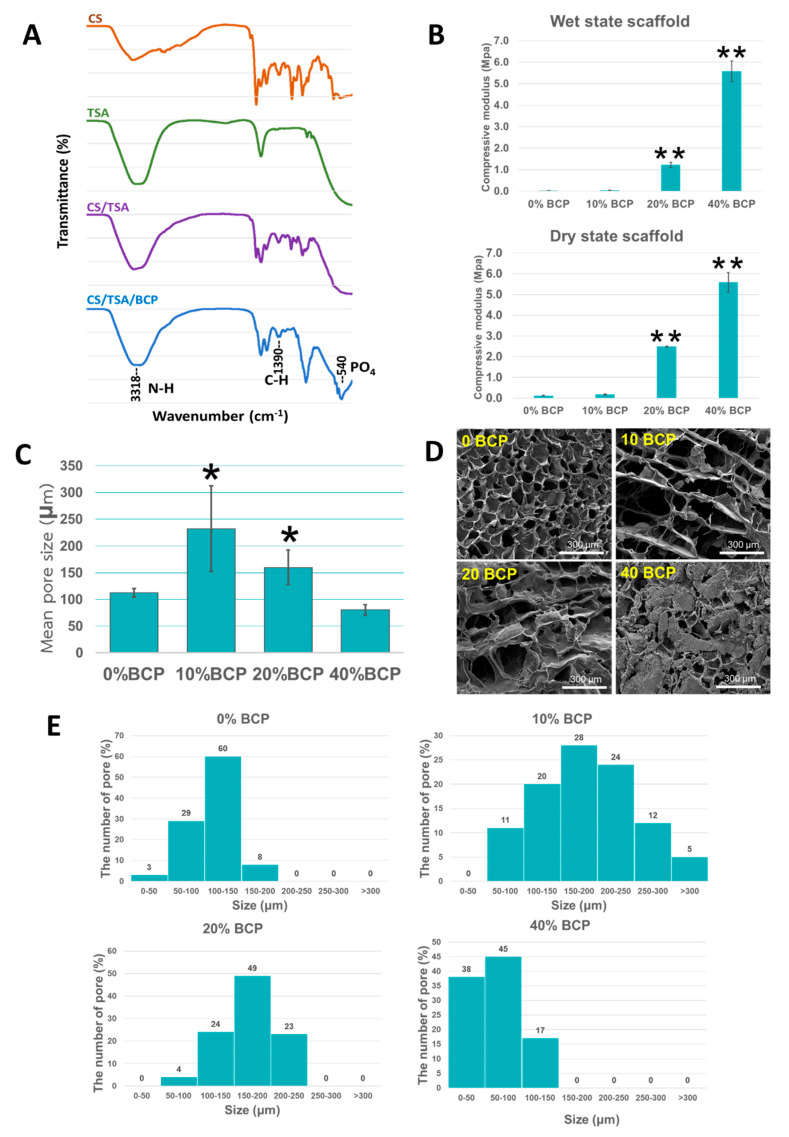
Physical characterization of CS/BCP/TSA scaffolds in various BCP proportions. (**A**) FT-IR spectra of CS/BCP/TSA scaffolds showed the absorption peak at 540, 600, 1390, and 3318 cm^−1^, representing each scaffold compartment. (**B**) With the increase in BCP proportion, the compressive strength of the scaffolds in the wet state was increased. (**C**) The mean pore size of CS/BCP/TSA scaffolds in various BCP proportions. (**D**) The SEM surface morphology of CS/BCP/TSA scaffolds in various BCP proportions. (**E**) The pore size distribution of CS/BCP/TSA scaffold in various BCP proportions. Values are defined as mean ± SD. * *p* < 0.05; ** *p* < 0.01.

The fabrication method and gross scaffold structure are shown in Figure 1A,B. The CS/BCP/TSA scaffolds with cylindrical shapes were successfully constructed by the freeze-drying technique. The morphology of scaffolds in different proportions of BCP was shown in Figure 1B. The BCP microparticles were homogeneously blended with the scaffold structure. Fourier-transform infrared (FTIR) spectra of CS, TSA, CS/BCP, and CS/BCP/TSA scaffolds were presented in Figure 2A. In the CS/BCP/TSA scaffolds sample, C-H bending appears at the FTIR absorption peak at 1390 cm^−1^, indicating the presence of CS. In comparison, the absorption peak at 540 and 600 cm^−1^ (between the ceramic and polymer matrix) demonstrated characteristics of PO_4_ bands of BCP. Moreover, successful TSA incorporation in the bio-ceramic matrix was confirmed when applying an absorption peak at 3318 cm^−1^ to N-H stretching of TSA.

With the addition of BCP, the compressive strength of the scaffolds was increased in both wet and dry states compared to the pure CS scaffold (Figure 2B). Physiologically, the scaffold for bone regeneration was always exposed to body fluids in its clinical use. Therefore, the mechanical strength evaluated after fluid immersion more closely mimics the stage of the scaffold in vivo. Scanning electron microscope (SEM) images showed an interconnected porous network in the scaffold of all groups except for the 40% BCP group, which presented low porosity and dense structure. Furthermore, the BCP particles were homogeneously distributed on the scaffold pore’s wall (Figure 2D). The average pore sizes of the scaffolds were 110 ± 6, 232 ± 160, 160 ± 52, and 88 ± 10 μm for 0%, 10%, 20%, and 40% proportion of BCP, respectively (Figure 2C). A high degree of pore size distribution was presented only in 10% and 20% BCP groups (Figure 2E).

**Figure 3:**
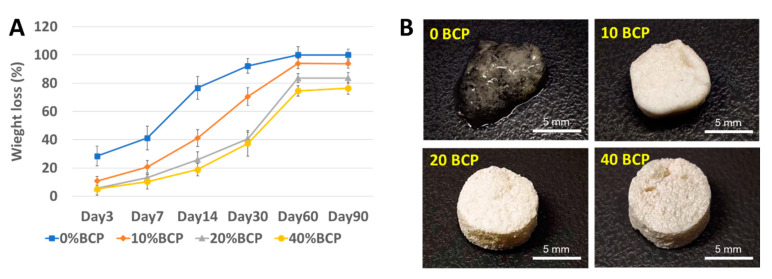
The enzymatic degradation behavior of CS/BCP/TSA scaffolds. (**A**) The weight changing of CS/BCP/TSA scaffolds at the time intervals of 1, 3, 7, 14, 30, and 60 days. (**B**) The gross structure of CS/BCP/TSA scaffolds before frizzed dry, at 30 days after enzymatic degradation.

The degradation behavior of the scaffolds in a solution containing lysozyme showed that pure CS scaffold degraded very fast by losing more than 80% of its original weight within 30 days. By increasing the BCP proportion, the scaffolds could maintain an interconnected porous structure while dramatically reducing the degradation rate (Figure 3A,B). From all physical and chemical characteristics, the 20% BCP scaffold was used for subsequence experiments.

### 2.2. The Scaffolds Demonstrate Cytocompatibility and Facilitate Cell Attachment

**Figure 4:**
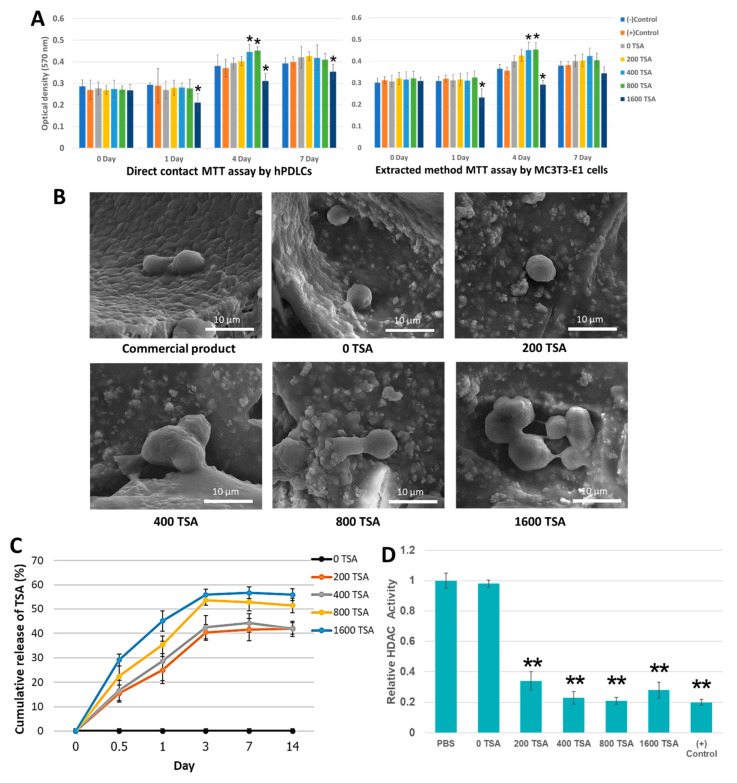
Biological properties of CS/BCP/TSA scaffolds in various TSA concentrations. (**A**) Cytocompatibility of CS/BCP/TSA scaffolds in various TSA concentrations by direct contact method with MTT assay. (**B**) SEM microphotographs of the hPDLCs cultured on CS/BCP/TSA scaffolds in various TSA concentrations. (**C**) Cumulative release curve of TSA. (**D**) The HDAC activity of hPDLCs treated with conditioned media of CS/BCP/TSA scaffolds. Values are defined as mean ± SD. * *p* < 0.05; ** *p* < 0.01.

The MTT assay was used to evaluate the effect of the synthesized scaffolds on hPDLCs and MC3T3-E1 cell viability for direct contact and extracted method, respectively, shown in Figure 4A. Both methods showed a similar trend of the results, which indicated that all samples positively affected hPDLCs and MC3T3-E1 cell viability. Moreover, the results clearly showed that the number of viable cells significantly increased in 400 and 800 nM of TSA-containing scaffolds after four days of culturing. However, 1600 nM of TSA-containing scaffolds significantly decreased the cell viability at all time points, indicating cytotoxicity. SEM evaluated the attachment and morphology of hPDLCs culture for 48 h on the samples. hPDLCs exhibited good adhesion on the scaffold surface of all groups and a positive control sample (Figure 4B). Interestingly, the cells seeded on a higher concentration of TSA-containing scaffolds displayed numerous pseudopod protrusions to contact adjacent cells.

### 2.3. Incorporated TSA Was Confirmed to Be Released and Stay Bioactive

To test the bioactivity of TSA in the scaffolds, TSA concentrations were set to 0, 200, 400, 800, and 1600 nM and measured at predetermined time points. Figure 4C showed a linear increase in TSA cumulative release during the first five days. A similar trend of initial rapid release of TSA was found within 24 h, followed by gradual release of up to 50% within three days. After 5 d, the TSA was completely released from the scaffolds. The cumulative releases of TSA were approximately 40%, 42%, 54%, and 57% in 200, 400, 800, and 1600 nM groups, respectively. The HDAC activity in the nuclear extracts of hPDLCs incubated with supernatant medium from the scaffold with TSA was four to five-fold lower than in control (Figure 4D). TSA concentration as low as 200 nM was able to inhibit HDAC activity. However, no significant difference in HDAC activities was observed among all TSA-incorporated groups. This result confirmed that TSA released from the scaffolds could inhibit HDAC activity of hPDLCs.

### 2.4. The Scaffold Induces Expression of Osteogenic Genes but Not Inflammatory Related Gene

**Figure 5:**
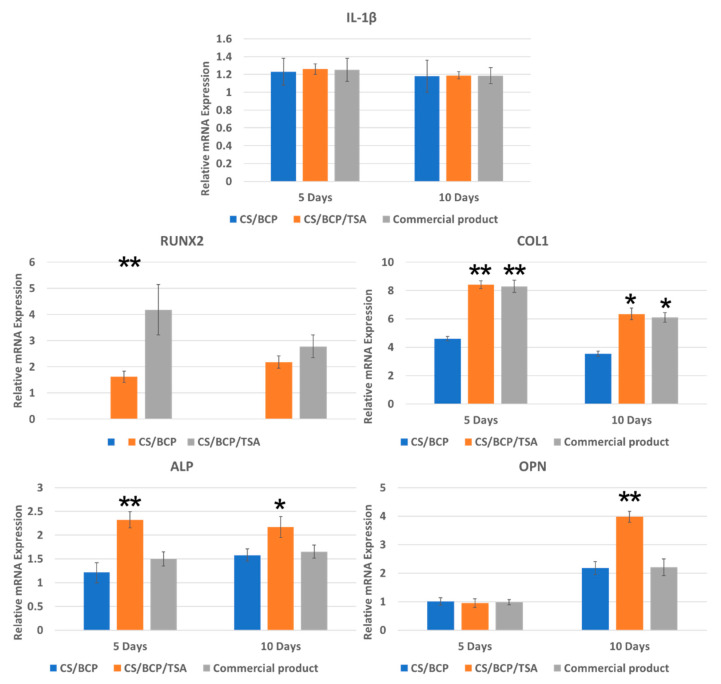
Expression of inflammatory and osteogenic genes of hPDLCs after cultured with CS/BCP/TSA scaffolds for 5 and 10 days, compared with CS/BCP without TSA loading and commercial bone graft product. Values are defined as mean ± SD. * *p* < 0.05; ** *p* < 0.01.

The influence of CS/BCP/TSA scaffolds on inflammation and osteoblast differentiation was investigated by observing gene expression for 5 and 10 days. The results confirmed that the newly fabricated scaffolds and commercial bone graft do not stimulate interleukin-1 beta (IL-1β), an inflammatory-related gene, compared to the negative control. In contrast, most osteoblast-related genes, including runt-related transcription factor 2 (RUNX2), type I collagen (COL1), alkaline phosphatase (ALP), osteopontin (OPN) were significantly induced by CS/BCP/TSA scaffold. After five days of culture, RUNX2, a master gene controlling osteoblast differentiation, showed a marked increased expression. OPN, late markers of differentiated osteoblasts, was highest after ten days of culture. COL1, the most abundant extracellular matrix protein in bone, and ALP, an early marker for osteoblast differentiation, were significantly upregulated at both time points (Figure 5).

### 2.5. CS/BCP/TSA Scaffold Promote Excellence Bone Regeneration In Vivo

**Figure 6:**
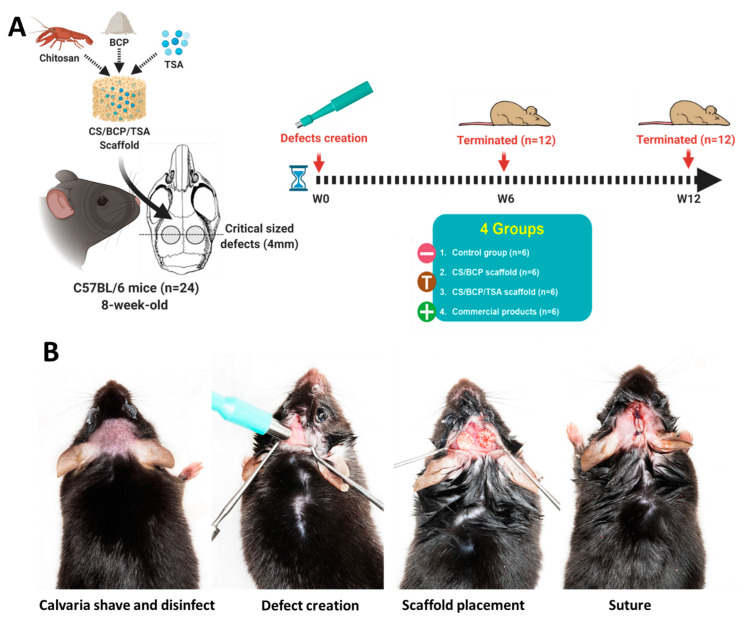
Mouse calvaria defect model. (**A**) A schematic illustration of animal experiments. (**B**) Surgical steps: Calvaria shave and disinfect, defect created by biopsy punch, scaffold placement, and wound closure by suture.

**Figure 7:**
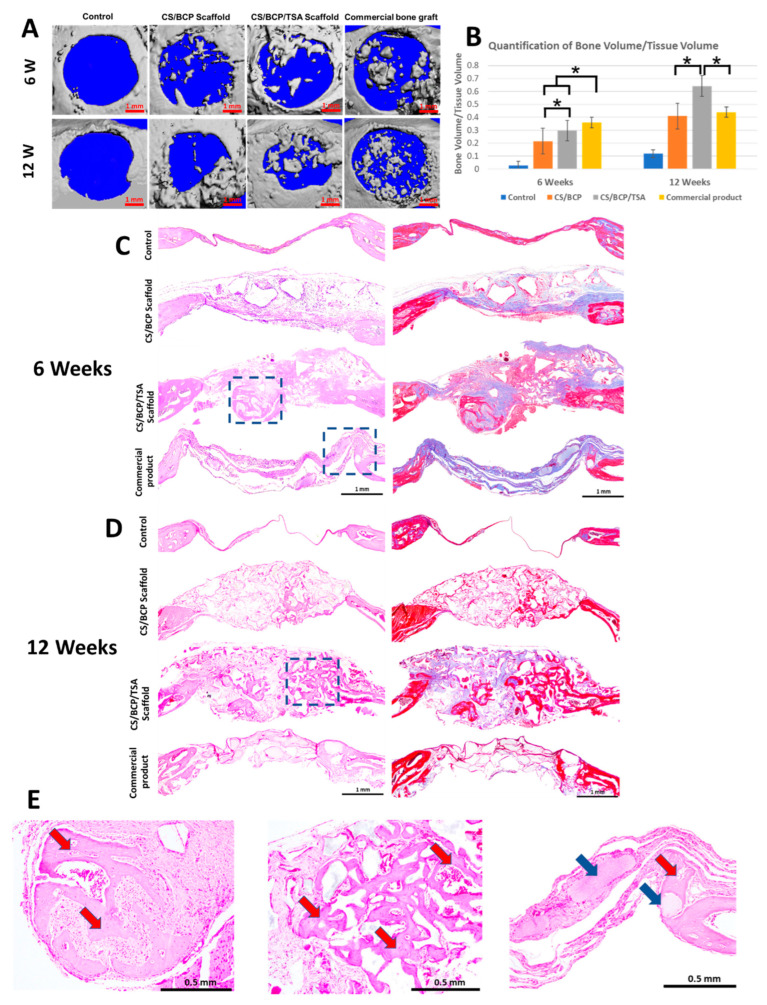
In vivo bone regeneration potential of CS/BCP/TSA scaffolds. (**A**) Micro-CT-based 3D images of new bone formation (Scale bar—1 mm). (**B**) Quantification of BV/TV of CS/BCP/TSA scaffolds, compared with CS/BCP without TSA loading and commercial bone graft product at 6 and 12 weeks after implantation. (**C**,**D**) By H&E and Masson’s trichrome staining, histological view of defects implanted with scaffold at 6 and 12 weeks after implantation, low magnification (4×), scale bar—1 mm. (**E**) High magnification (40×), from left to right panels, showing the trabecular bone ingrowth in CS/BCP/TSA scaffolds both 6 and 12 weeks (red arrows), while commercial graft group remains incompleted BCP resorption (blue arrows), scale bar—0.5 mm, * *p* < 0.05.

The animal procedures are shown in Figure 6A,B. Micro-CT was used to evaluate the mineralized content in mouse calvarial defects. At six weeks post-implantation, 3D constructed images showed hard tissue formation in newly fabricated scaffolds groups and the commercial product group. In contrast, hard tissue formation in the defects for the sham group was not detected. After 12 weeks, new hard tissue formation was seen throughout the defects, especially the in-growth of new bone from the periphery of the defect in the CS/BCP/TSA scaffold group compared to other groups (Figure 7A). Although quantification of bone volume to total volume fraction (BV/TV) demonstrated that the commercial product group has the highest BV/TV at 6-week, CS/BCP/TSA scaffold group showed a marked increase in the amount of BV/TV at 12-weeks (Figure 7B).

The histological analysis of sections stained with H&E and Masson’s trichrome was shown in Figure 7C,D. At six weeks, the new bone formation starts from the peripheral region of all implanted groups. The CS scaffold groups showed thick connective tissue with osteoid deposition. The trabeculae-like structure implied mature bone was found in the defect implanted with CS/BCP/TSA scaffold at as early as six weeks. New bone formation in growth from the defect’s periphery seamlessly continues with the pre-existing bone was seen in the CS/BCP/TSA scaffold group (Figure 7E). Commercial bone graft implantation showed the incomplete degradation of BCP particles surrounding fibrous tissue. At 12 weeks. The negative control group showed a thin layer of connective tissue with no bone formation at either time point.

## 3. Discussion

In the present study, highly porous CS-based scaffolds were fabricated using a freeze-drying technique. First, we worked to identify the proportion of BCP which showed the most suitable biomechanical properties. The results indicated that the physicochemical characterizations of CS scaffold (pore size, degradation rate, and mechanical strength) were significantly improved by incorporating BCP. Previous studies have shown that heterogeneous pore sizes in the range of 100–325 μm with a high degree of distribution are optimal for bone scaffolds [31]. The optimal proportion of interconnected pores should be used somewhere in the range of 100–200 μm for maximum cell adhesion and proliferation. In contrast, a faster rate of neovascularization is achieved through the small fraction of the larger porous structure (>300 μm). The degradation rate should be equal to regenerated bone [32]. Additionally, optimal compressive strength of bone scaffold is achieved when given in the range of 1–12 Mpa for the provided 3D matrix, which allows the proliferation of osteoinducible cells [33,34]. All our testing results proved that CS-based scaffolds with 20% of BCP meet or exceed the minimum requirements of physicochemical performance for bone tissue engineering scaffolds. This phenomenon is attributed not only to the formation of a strong network penetration of BCP particles into the surface of CS networks but also to strong ionic interaction between PO_4_^−3^ in BCP with NH^3+^ in CS [35,36].

In response to environmental and metabolic activities, epigenetic processes regulate the modulation of gene expression via microRNA regulation, DNA methylation, or histone modification [28,37]. Furthermore, previous studies have proven that TSA, an epigenetic modifier has a beneficial effect on bone repair and regeneration. Li et al. reported that TSA could promote osteogenic differentiation of mesenchymal stem cells by inhibiting the nuclear factor-κB [26]. Additionally, our studies confirmed that TSA has the ability to induce osteogenic differentiation of hPDLCs and demonstrates a potential application of TSA for bone regeneration therapy [25,29]. The results show that TSA release profile can be divided into two phases. Phase 1: TSA had an initial burst within 24 h. Phase 2 (24 h–3 d), TSA sustained release from the scaffolds at 55%. These phenomena might result from the weak hydrogen bond between the hydrophilic part of the TSA molecule and the hydroxyl group of CS. Notably, the total amount of TSA released from 800 nM TSA scaffold was calculated to be 435 ± 16 nM, which showed excellent biocompatibility and enhanced hPDLCs and MC3T3-E1 cell proliferation, whereas the 1600 nM TSA scaffold showed toxicity with the cells. These data corresponded with our previous study that the optimal concentration of TSA to induce osteogenic differentiation without cytotoxicity is 400 nM [29]. The CS/BCP scaffolds act as a suitable carrier, providing a sustained release ability for TSA. The CS/BCP/TSA scaffold with 20% of BCP and 800 nM of TSA was determined to be the optimal proportion to provide mechanical and biological properties to support bone formation.

The most important strength of the study was that it was the first experimental evidence for incorporating the HDAC inhibitor molecule into the cell-free bone regeneration scaffold. Previous studies often used epigenetic reprogramming cells for cell-based scaffold regeneration [38,39]. Moreover, this study was conducted to cover all necessary aspects, including scaffold preparation, in vitro physical and biological properties evaluation, and in vivo bone regeneration. One limitation of this study is the lack of evidence about the optimal exposure time of TSA for maximizing bone regeneration. Compared to the bone healing processes, the first three days in the healing period might be too early for inducing the interplay of osteoprogenitor cells. This phenomenon is explained by weak non-covalent immobilization between TSA molecules and the polymeric chain of CS. Evidence has shown that microfabrication techniques, such as 3-dimensional printing or electrospinning and using a microsphere release system, can enable drug molecules to sustain release in sequential manners [40,41,42]. Further studies should be conducted to modify the fabrication procedure and TSA loading method, resulting in a controlled release fashion.

At six weeks, our in vivo results demonstrated a significant increase in BV/TV in the defects implanted with CS/BCP scaffold with or without TSA and commercial bone graft. The commercial bone graft showed the highest BV/TV at this time point. These phenomena might result from incompleted degradation of BCP, which is a major mineralized content in the scaffold. This evidence was clearly seen in histology results. Notable increases in bone formation were found in scaffold loaded with TSA at the 12-week time interval. Remarkably, histological analysis reveals trabeculae-like structure seamlessly continues with pre-existing bone in the defects implanted with CS/BCP/TSA scaffold at both time points. This result corresponded with the previous study [43] that TSA is a potent osteogenic inducer and can enhance bone formation in an animal model. The in vivo results confirmed that the CS/BCP/TSA scaffold possesses excellent osteogenic bone tissue engineering potential.

## 4. Materials and Methods

### 4.1. Fabrication of CS/BCP/TSA Scaffold

First, 4% *w*/*v* of medium-molecular-weight chitosan (Mw: 250 kDa, particle size: 0.5 mm, ≥90% deacetylated, Marine Bio Resources, Samutsakhon, Thailand) was dissolved in succinic acid solution and stirred overnight at 60 °C. The different amounts of BCP (Particle size: 0.1–0.5 mm, MTEC, Pathumthani, Thailand), 10%, 20%, 40% *w*/*w*, and TSA (Sigma-Aldrich, Oakville, Canada) 200, 400, 800, 1600 nM were added into chitosan (CS) solution. To prepare a CS hydrogel, the 1-(3 Dimethylamino-propyl)-3 ethylcarbodiimide hydrochloride (EDC)/N-Hydroxysuccinimide (NHS) molar ratio was set as 1:1. Finally, hydrogels were freeze-dried at −50 °C for 48 h to obtain CS/BCP/TSA scaffold.

### 4.2. Culture of Primary hPDLCs

With informed consent, the hPDLCs were obtained from the extracted healthy third molars of young individuals aged 18–25-year-old. All procedures were performed under the approval of the Ethical Committee of the Faculty of Dentistry, Chulalongkorn University, Thailand (Approval Number: HREC-DCU 2020-106). This study was performed in accordance with the Declaration of Helsinki. The third molars were washed, and the soft tissue attached to the cervical area was removed. Then the periodontal ligament attached to the central one-third of the root surface was carefully scraped off. Tissue samples were seeded in a culture medium (10% FBS, 1% L-Glutamine, 1% antibiotics in DMEM (Gibco, Thermo Fisher Scientific, Inc., Waltham, MA, USA)). The cells were incubated at 37 °C humidified atmosphere with 5% CO_2_. The hPDLCs in the third to fifth passage were used in this study.

### 4.3. Physical and Chemical Characterization of the Scaffolds

The CS/BCP/TSA scaffolds were prepared into a cylinder shape (10 × 8 mm). FTIR was conducted using a Perkin-Elmer spectrometer to analyze the chemical contents of the scaffolds in the standard frequency range (4000–400 cm^−1^). The surface morphologies of the scaffolds were observed by SEM and evaluated by image visualization software (Image J version 1.53, NIH, Bethesda, MD, USA). The mean pore size and pore size distribution were calculated on random 10 mm^2^ of SEM images based on 100 pores. All measurements were performed in triplicate.

### 4.4. In Vitro Degradation of the Scaffolds

Degradation tests were conducted in PBS containing 1.5 μg mL^−1^ lysozyme (hen egg-white, Sigma-Aldrich, Oakville, Canada) at 37 °C. The degraded scaffolds were freeze-dried again and then the weight changing at the time intervals of 1, 3, 7, 14, 30, and 60 days was calculated.

### 4.5. Mechanical Properties

The compressive modulus of the scaffolds in both the dry and wet state were determined using a universal testing machine (Instron Model 8500, Instron Corp., Canton, MA). The compression rate was set at 2 mm/min, and the load was applied until the samples were pushed to approximately 80% of their original shape. All samples were performed in triplicate.

### 4.6. Cellular Biocompatibility

According to the MTT assay, the viability was measured on both hPDLCs and MC3T3-E1 cells. MC3T3-E1 cells were purchased from Biomedia (Nonthaburi, Thailand). The CS/BCP/TSA scaffolds and commercial bone graft products (Osteon III collagen, Dentium, Seoul, Korea) were sterilized under UV light for 30 min. For the direct contact test, hPDLCs 5 × 10^3^ cells/well were cultured on a well plate for 24 h, then placed a scaffold on top of the monolayer cells and incubated for 0, 1, 4, and 7 days. For the indirect contact test, a small piece of scaffold (0.2 g/mL) was immersed in culture medium for 24 h at 37 °C. The supernatant obtained was applied to the monolayer culture of MC3T3-E1, incubated for 0, 1, 4, and 7 days, then added 10 µL of MTT per well with a subsequent incubation for 2 h. and measured the absorbance at 570 nm. The cells cultured without scaffold served as a control.

### 4.7. In Vitro Release and Bioactivity of TSA

In vitro release of TSA was determined by incubating 20 mg of scaffolds in 25 mL of PBS separately maintained in the shaker (60 rpm) at the physiological condition for 28 days. At the time intervals of 1, 3, 7, 28 days, 1 mL of the samples were collected and analyzed using at 280 nm with a using UV-Vis spectrophotometer. A standard curve of pure TSA in a standard solution ranging from 0 to 1600 nM was constructed. The cumulative release of TSA from the CS/BCP/TSA scaffolds at each time interval was calculated and expressed as a percentage of initial loading.

The bioactivity of TSA that was released from CS/BCP/TSA scaffolds was evaluated by determining its HDAC activity with the fluorometric HDAC Activity Assay kit (Abcam, Cambridge, MA, USA). Briefly, hPDLCs were treated with 1 mL of supernatant collected from the scaffolds and incubated for 72 h. The cell lysates were incubated with substrate peptide for 30 min on a microtiter plate. Then, the developing solution was added to each well for a further 20 min. The reaction was stopped by adding the stop buffer, and the fluorescence intensity at Ex/Em  =  350/460 nm was measured in a fluorescence plate reader.

### 4.8. Cells Attachment and Morphology

The attachment and morphology of hPDLCs in scaffolds at 48 h were observed by using SEM. The 10^5^ hPDLCs were seeded onto a scaffold and incubated for 48 h. Before observing cell adhesion, the samples were rinsed three times with PBS to eliminate any unattached hPDLCs and fixed in 2.5% glutaraldehyde. Then, samples were dehydrated in a gradient ethanol series and dried at room temperature.

### 4.9. Expression of Inflammatory and Osteogenic Genes

To estimate the effect of CS/BCP/TSA scaffolds on the expression of the inflammatory-related gene and osteoblast-related genes by hPDLCs, quantitative real-time PCR was performed (Appendix for details of experiment). hPDLCs were incubated with CS/BCP/TSA scaffolds and commercial bone graft products for 5 and 10 days in osteogenic medium. PCR primer for interleukin-1 beta (IL-1β), runt-related transcription factor 2 (RUNX2), type I collagen (COL1), alkaline phosphatase (ALP), osteopontin (OPN), (Sigma-Aldrich, St. Louis, MO, USA) were used to screen the inflammatory-related gene and osteoblast-related genes, which were normalized against the expression level of the GAPDH control. Details of the PCR primers used are described in Table 1. All samples were performed in triplicate.

### 4.10. In Vivo Bone Regeneration

The animal procedure, modified from our previous study [44], was approved by Chulalongkorn University Animal Care and Use Committee (CU-ACUC), Thailand (Animal Use Protocol No. 2073027). This study is reported in accordance with ARRIVE guidelines. The sample size was calculated based on the data from our previous similar study using G*Power program by setting α-error = 0.05, power = 0.8, and effect size = 0.8. Twenty-four 8-week-old C57BL/6 mice (age: 8 weeks, weight: 20–35 g) were anesthetized with intraperitoneal injection of Zoletil100 (40 mg/kg) and xylazine (5 mg/kg). Two bilateral full thickness calvarial defects (4 mm in diameter) were created using a biopsy punch (Stiefel, GSK, NC, USA). Mice were simple randomly divided into four groups as follows: (1) CS/BCP scaffold, (2) CS/BCP/TSA scaffold, (3) commercial bone graft products as a positive control, and (4) empty defects as a negative control. The mice were weighed daily and monitored for signs of illness, infection, pain, or distress twice daily for the first two post-operative days. Following this, they were weighed and checked daily until post-operative day 7. They were then weighed and monitored on a weekly basis. Post-operatively, mice were housed singularly and transferred to a warming cabinet overnight for recovery. Carprofen (10 mg/kg) was administered subcutaneously twice daily for 4 days post-operatively for analgesia. After being euthanized at 6 weeks and 12 weeks, samples were excised and fixed in 10% buffered formalin. The mineral density and the morphology of the defects (n = 12 per group) were evaluated by a micro-CT scanner (SCANCO Medical AG, μCT 35, Switzerland). The samples were decalcified and embedded in paraffin and were then cut along the larger diameter of the defect. Sections at ten μm each were stained with hematoxylin and eosin (H&E) and Masson’s Trichrome.

### 4.11. Statistical Analysis

The statistical analysis was performed with SPSS 23 (IBM, Armonk, NY, USA) using the One-Way Analysis of Variance (ANOVA) test for both in vitro and in vivo studies. Significant differences were indicated as: * (*p* < 0.05) for significant and ** (*p* < 0.01) for highly significant differences.

## 5. Conclusions

Our study demonstrated that highly porous CS/BCP/TSA scaffolds could be constructed using a simple freeze-drying technique. The addition of BCP to the CS improved mechanical properties and delayed the degradation rate. Scaffolds with 20% of BCP and 800 nM of TSA showed excellent physicochemical structure, biocompatibility, and sustained release of TSA for up to three days. TSA released can inhibit histone deacetylase enzyme in hPDLCs resulting in upregulating most osteoblast-related genes. The CS/BCP/TSA scaffolds’ excellent bone regeneration capability was confirmed in the mice calvarial model. This study provided CS/BCP/TSA scaffolds as an up-and-coming candidate for bone tissue engineering applications.

## Figures and Tables

**Table 1:** The primer sequences of the upstream and downstream primers for these mRNA analyses (5′ to 3′).

**IL-1β**	TGGACCTTCCAGGATGAGGACA	GTTCATCTCGGAGCCTGTAGTG
**COL-1**	GTGCTAAAGGTGCCAATGGT	ACCAGGTTCACCGCTGTTAC
**RUNX2**	CCCCACGACAACCGCACCAT	CACTCCGGCCCACAAATC
**ALP**	CGAGATACAAGCACTCCCACTTC	CTGTTCAGCTCGTACTGCATGTC
**OPN**	AGGAGGAGGCAGAGCACA	CTGGTATGGCACAGGTGATG

## Data Availability

The authors confirm that the data supporting the findings of this study are available within the article. Raw data that support the findings are available from the corresponding author, upon reasonable request.
